# Satisfaction Resilience in Virtual Home Health Care for Chronic Disease Management: Lifestyle-Integration Motivation and Functional Equivalence Beliefs in a Cross-Sectional Study

**DOI:** 10.3390/healthcare14162484

**Published:** 2026-08-11

**Authors:** Ahmed Alqheedan, Saleh Alzughaibi, Eman Alanazi, Mohammed Alsahli, Amani Othman, Rasha Alhazzaa, Mansour Almanaa

**Affiliations:** 1 Department of Health Informatics, College of Health Sciences, Saudi Electronic University, Riyadh 13316, Saudi Arabia; a.alqheedan@seu.edu.sa (A.A.); e.alanazi@seu.edu.sa (E.A.); m.alsahli@seu.edu.sa (M.A.); 2 Department of Public Health, College of Health Sciences, Saudi Electronic University, Riyadh 13316, Saudi Arabia; a.othman@seu.edu.sa; 3 Department of Health Informatics, College of Health Sciences, Saudi Electronic University, Dammam 32256, Saudi Arabia; r.alhazzaa@seu.edu.sa; 4 Radiological Sciences Department, College of Applied Medical Sciences, King Saud University, Riyadh 12372, Saudi Arabia; malmanaa@ksu.edu.sa

**Keywords:** virtual home health care, telehealth, chronic disease management, patient satisfaction, satisfaction resilience, functional equivalence, healthcare service design

## Abstract

**Highlights:**

**What are the main findings?**

- Most patients with multiple Virtual Home Health Care barriers still reported high satisfaction, showing that satisfaction can be preserved even under barrier exposure.
- Lifestyle-integration motivations and perceived functional equivalence with in-person care distinguished resilient patients from vulnerable patients more clearly than demographic characteristics.

**What are the implications of the main findings?**

- Virtual Home Health Care programs should move beyond barrier reduction alone and actively strengthen satisfaction resilience among patients managing chronic diseases.
- Communication and service-design strategies should emphasize how virtual care fits into daily life and delivers care that patients perceive as functionally comparable to in-person visits.

**Abstract:**

**Background/Objectives**: Virtual home health care (VHHC) is increasingly used to support chronic disease management, yet patient satisfaction is often examined through a linear barrier-focused lens, in which more barriers are assumed to produce lower satisfaction. This study introduced a satisfaction resilience framework to identify factors associated with preserved satisfaction among barrier-exposed VHHC users. **Methods**: This cross-sectional analysis included 517 adults with chronic diseases who had used VHHC services and were recruited across five regions of Saudi Arabia. Patients were classified by barrier load and overall satisfaction into four groups: Resilient, Vulnerable, Comfortable, and Disengaged. Group differences were examined using one-way ANOVA, chi-square tests with Cramér’s V, point-biserial correlations, and a linear probability model restricted to high-barrier patients. **Results**: Overall, 167 patients (32.3%) reported high barrier exposure. Among them, 109 (65.3%) were classified as Resilient and 58 (34.7%) as Vulnerable, despite statistically equivalent barrier counts. Resilient patients reported higher motivation count, provider trust, perceived effectiveness, and functional equivalence with in-person care. Lifestyle-integration motivations, particularly avoiding work absence and waiting rooms, differentiated Resilient from Vulnerable patients, whereas access-related motivations did not. Perceived functional equivalence showed the largest between-group effect. In the high-barrier subsample, motivation count and provider trust were independently associated with Resilient status, while demographic variables were not significantly associated with it. **Conclusions**: Satisfaction with VHHC among chronic disease patients is not determined by barrier exposure alone. Service-design and communication strategies that strengthen lifestyle integration, functional equivalence beliefs, and provider trust may help healthcare teams support satisfaction resilience among patients who experience barriers to virtual care.

## 1. Introduction

Saudi Arabia is undergoing a major digital health transformation under the Vision 2030 Health Sector Transformation Program. The Seha Virtual Hospital, launched in February 2022, is officially recognized by Guinness World Records as the world’s largest virtual healthcare provider [1]. It currently supports more than 242 hospitals, provides services across 48 main specialties and 68 sub-specialties, and has an annual operational capacity exceeding 597,000 beneficiaries [2]. In 2025 alone, the hospital recorded more than 16 million virtual appointments and consultations, spanning nationwide services from routine outpatient care to time-critical telestroke management during mass-gathering seasons [3,4,5]. Among the services driving this expansion is Virtual Home Health Care (VHHC), a model in which patients receive consultations, medication reviews, and chronic disease monitoring through digital platforms while remaining at home [6,7,8].

Chronic disease is common among Saudi adults. In a recent national telephone survey, hypertension and diabetes were the most commonly reported chronic conditions, with estimated prevalences of 16.1% and 13.0%, respectively, and roughly one in ten adults reported two or more chronic conditions [9]. The long-term management burden of diabetes, hypertension, asthma, cardiovascular disease, and cancer makes this population a primary beneficiary of VHHC. International evidence supports the model’s clinical effectiveness, and systematic reviews consistently report patient satisfaction levels comparable with in-person care and outcome equivalence across multiple disease groups [10,11,12,13]. Yet patients also report barriers, including concerns about diagnostic accuracy, perceived care quality, privacy, and consultation recording [6,14]. The dominant analytical paradigm in this literature treats these barriers as predictors that should subtract from satisfaction in linear fashion: more barriers, less satisfaction. Almost every regression-based satisfaction study assumes this uniform relationship [15,16,17], and the J.D. Power 2024 U.S. Telehealth Satisfaction Study ranked telehealth barriers by prevalence without examining the variance in their effects on satisfaction [18].

### 1.1. The Limits of the Linear Barrier–Satisfaction Assumption

The linear assumption may be empirically and clinically incomplete. Patients with the same barrier profiles may differ substantially in satisfaction outcomes, suggesting that additional motivational or psychological factors may be associated with preserved satisfaction under barrier exposure. The resources associated with this pattern remain unexamined. This is not a minor observation. If certain motivational or psychological resources reduce the impact of barriers, the practical implication is substantial. VHHC program planners seeking to improve satisfaction would be better served by strengthening these resources than by attempting to eliminate barriers that may not be removable in the short term, such as concerns about diagnostic accuracy in audio-visual consultations. The orientation shifts from barrier elimination, which is partial and slow, to resilience building, which can be achieved through communication and service design.

### 1.2. From Barrier Reduction to Resilience Building

The concept of resilience, defined as the capacity to maintain positive functioning despite adversity, is well established in chronic disease self-management [19,20,21,22,23], mental health [24], and organizational behavior. Within healthcare, resilience research has typically focused on patients’ coping with disease, treatment burden, and the psychological consequences of illness. To the best of our knowledge, its application to telehealth satisfaction has not been explored. We propose that the concept of resilience can be effectively applied to the digital health context. The present study applies and adapts this concept to the service evaluation context. We recognize that traditional resilience research requires longitudinal measurement to track recovery over time. Because the present design is cross-sectional, we define satisfaction resilience as a state rather than a process: the maintenance of high satisfaction despite multiple concurrent barriers. We acknowledge that the temporal dynamics of resilience cannot be examined from a single observation. The term “resilience” is therefore used descriptively, to denote preserved satisfaction under barrier exposure rather than recovery or adaptation over time. Specifically, *satisfaction resilience* is defined as maintaining high overall satisfaction (≥4 on a 5-point scale) while simultaneously reporting multiple service barriers (≥2 endorsed). Combining barrier load with satisfaction creates a 2 × 2 typology with four groups: Resilient (high barrier, high satisfaction), Vulnerable (high barrier, low satisfaction), Comfortable (low barrier, high satisfaction), and Disengaged (low barrier, low satisfaction). The ≥2 barrier threshold was selected to distinguish isolated concerns from concurrent barrier exposure, which is more consistent with the proposed resilience framework. The E12 ≥4 threshold was selected because responses of 4 or 5 correspond to agreement or strong agreement with the overall satisfaction item. These thresholds were used for conceptual classification rather than clinical diagnosis. This framework shifts the analytical focus from “who is satisfied?” to “who remains satisfied under barrier exposure, and what factors are associated with preserved satisfaction?”

### 1.3. Motivation as a Candidate Resilience Resource

The Capability, Opportunity, Motivation–Behavior (COM-B) model [25] proposes that motivation can sustain health behavior even when opportunity is constrained. Similarly, the Health Belief Model [26] suggests that perceived benefits can outweigh perceived barriers when they are sufficiently important. Recent COM-B applications to digital health adoption among older adults with chronic diseases have shown that motivational factors override opportunity constraints in self-management settings [27]. However, the effect of motivation on resilience in VHHC satisfaction has not been examined.

A separate but related question concerns the different types of motivations behind VHHC use. Users commonly report several reasons for choosing VHHC, such as speed of access, avoidance of transportation, avoidance of waiting rooms, and avoidance of work absence. These motivations are usually reported together rather than classified into distinct categories. We argue that they fall into two qualitatively different categories. *Access motivations* (speed of access, no transportation needed) describe the logistical advantages of remote care; they explain why a patient might choose VHHC for a remote consultation. *Lifestyle-integration motivations* (avoiding work absence, avoiding waiting rooms) describe how VHHC fits into the daily routines and competing obligations of working adults; they explain why a patient might prefer VHHC over time. The two categories may differ considerably in how they support resilience. Access motivations are common among many users and may not clearly distinguish satisfied users from dissatisfied users. Lifestyle-integration motivations, by contrast, reflect a structural fit between VHHC and the patient’s daily life. Patients who endorse these motivations may perceive a stronger fit between VHHC and their daily routines, which may help explain why some remain satisfied when barriers are present.

### 1.4. Functional Equivalence as a Threshold Belief

Beyond motivation, perceived functional equivalence with in-person care has emerged as a central evaluation in the telehealth literature [12,15]. The belief that a virtual consultation achieves what an in-person visit achieves is structurally different from convenience or trust appraisals. It is an outcome judgment: the patient compares the result of the encounter, not the experience of it. Functional equivalence may also operate as a threshold belief: once a patient perceives that virtual care achieves what an in-person visit achieves, that perception may be associated with preserved satisfaction even under multiple barriers. However, this possibility has not been directly examined in VHHC populations.

### 1.5. Study Objectives and Research Questions

The overall aim of this study was to introduce and empirically examine a satisfaction resilience framework for VHHC among chronic disease patients in Saudi Arabia. To achieve this aim, the study pursued three specific objectives: (i) to characterize the distribution of patients across the four satisfaction resilience groups and to describe how these groups differ on demographic, motivational, and effectiveness variables; (ii) to identify the motivational and psychological factors that distinguish Resilient from Vulnerable patients among those facing equivalent barrier loads; and (iii) to determine whether demographic variables are associated with resilience group membership, both across the full sample and within the high-barrier subsample. These objectives were operationalized through the following three research questions, addressed in a sample of 517 chronic disease patients using VHHC in Saudi Arabia.

RQ1. What is the distribution of patients across the four satisfaction resilience groups, and how do these groups differ on demographic, motivational, and effectiveness variables?

RQ2. Among patients facing equivalent barrier loads, what motivational and psychological factors distinguish the Resilient from the Vulnerable group?

RQ3. Are demographic variables (gender, age, education, disease duration) associated with resilience group membership, and does this association persist within the high-barrier subsample?

The findings are intended to inform communication and service design strategies for the rapidly expanding Saudi VHHC infrastructure [1,2,28] and to provide a framework that can be applied across the multiple disciplines involved in digital home-based care delivery.

## 2. Materials and Methods

### 2.1. Study Design

This is an analysis of cross-sectional data originally collected by Elsayed and colleagues [6]. The original study examined VHHC effectiveness perceptions among adult chronic disease patients but did not analyze the multi-select motivation and barrier items. The present study uses these previously unanalyzed items together with the effectiveness data to develop and examine the satisfaction resilience framework. The two studies share the same sample but address distinct research questions and analyze different subsets of the variables.

### 2.2. Sample Size and Participants

The sample comprised 517 adult patients (≥18 years) with at least one chronic disease who had used VHHC services. The study was conducted at the national level in Saudi Arabia rather than in a single facility or region; recruitment invitations were circulated across the five administrative regions of the Kingdom (Central, Eastern, Western, Northern, and Southern), although the realized sample was concentrated in the Central Region (88%), as detailed below and discussed in the Limitations. A non-probability convenience sampling technique was used, in which participants who met the eligibility criteria and voluntarily responded to the online invitation were enrolled; no probability-based sampling frame or random selection was applied. Participants were recruited via an online questionnaire distributed across the five administrative regions of Saudi Arabia between March and June 2025. Recruitment was conducted through digital health platforms and healthcare facility networks; full details of the original recruitment procedure and response rate are reported in Elsayed et al. [6]. Chronic disease status was confirmed through self-report at questionnaire completion; participants indicated their primary chronic diagnosis as part of the survey instrument. The sample was predominantly female (*n* = 360, 69.6%), with the largest age group being 25–35 years (*n* = 204, 39.5%). Education distribution was as follows: high school or below (*n* = 69, 13.3%), bachelor’s degree (*n* = 315, 60.9%), and postgraduate degree (*n* = 133, 25.7%). The majority (*n* = 297, 57.4%) had lived with their chronic disease for more than five years. Geographic distribution was concentrated in the Central Region (88%); this concentration is addressed in the Limitations.

### 2.3. Study Instrument

The questionnaire included three parts. The demographic section captured gender, age, education, chronic disease type, disease duration, and region of residence. The effectiveness section comprised 11 Likert-scaled items (E1–E9, E11, E12) covering convenience, trust, access, communication, equivalence, and overall satisfaction. Each item was rated on a 5-point Likert scale anchored as follows: 1 = “strongly disagree,” 2 = “disagree,” 3 = “neutral,” 4 = “agree,” and 5 = “strongly agree,” with higher scores indicating more favorable perceptions. One additional audio-clarity item (E10) from the original 12-item instrument was excluded from the analysis dataset because of high redundancy with the visual equivalence item E9 (E9–E10 r = 0.708). The motivation and barrier section contained two structured multi-select items: motivations for using VHHC and barriers deterring use. The four motivation options were speed of access, no transportation needed, avoiding waiting rooms, and no work absence required. The four barrier options were accuracy doubt, quality concern, privacy worry, and recording concern. Respondents could select any combination of items. Responses were stored as Arabic semicolon-delimited strings corresponding to these fixed pre-specified options. Recoding into binary indicators (0 = not endorsed, 1 = endorsed) was performed using a deterministic string-matching process.

### 2.4. Resilience Group Classification

Two binary indicators were derived from the data. The barrier indicator was set to 1 if the patient endorsed two or more of the four barrier items (high-barrier) and 0 otherwise (low-barrier). The satisfaction indicator was set to 1 if the patient’s response to E12 (“Overall, I am satisfied with VHHC”) was 4 or 5 (high satisfaction) and 0 otherwise (low satisfaction). The cross-tabulation of these two binary indicators created the four resilience groups: Resilient (high barrier, high satisfaction), Vulnerable (high barrier, low satisfaction), Comfortable (low barrier, high satisfaction), and Disengaged (low barrier, low satisfaction).

### 2.5. Instrument Validity and Reliability

The questionnaire used in this study was the instrument developed and validated by Elsayed and colleagues [6], whose items were derived from the established telehealth and patient-satisfaction literature. The effectiveness items reflecting convenience, access, communication, provider trust, functional equivalence, and overall satisfaction were informed by prior validated telehealth satisfaction and evaluation frameworks, including the systematic review of telehealth patient-satisfaction scales by Du and Gu [15], the telehealth satisfaction evidence synthesized by Kruse et al. [10] and Nguyen et al. [17], and satisfaction measurement in chronic disease telehealth populations reported by Hendy et al. [16]. The multi-select motivation items (speed of access, no transportation needed, avoiding waiting rooms, no work absence) and barrier items (accuracy doubt, quality concern, privacy worry, recording concern) were adapted from the commonly reported drivers of and barriers to telehealth use documented in these sources and in the barrier-focused telemedicine literature [12,14,18]. The instrument was originally validated by Elsayed and colleagues through expert panel review, in which content and face validity were established by a panel of subject-matter experts prior to administration [6]. Internal consistency for the 12-item original scale was Cronbach’s α = 0.889; for the 11-item scale used in the present analysis, α = 0.870, indicating good internal consistency.

### 2.6. Statistical Analysis

Analyses were conducted using SAS v9.4 (SAS OnDemand for Academics) [29], with the significance threshold set at α = 0.05. Descriptive statistics (frequencies, means, standard deviations) were calculated for all variables. One-way analysis of variance with Tukey HSD post hoc tests was used to compare the four resilience groups on continuous variables, with η^2^ reported as the effect-size index. Chi-square tests of independence with Cramér’s V were used to compare endorsement rates of motivation and barrier items between Resilient and Vulnerable patients (the two high-barrier groups). Point-biserial correlations were calculated between each motivation item and resilience status within the high-barrier subsample. A linear probability model was used to examine the relationship of binary resilience status with motivation count, provider trust (E2), and four demographic variables within the 167 high-barrier patients to identify the unique predictors of resilience among the at-risk population. Cohen’s d was reported for between-group comparisons; effect-size conventions followed Cohen’s recommendations [30]. The decision to use a linear probability model rather than logistic regression was made to simplify coefficient interpretation in this descriptive context, where the binary outcome was approximately balanced (65/35) and predicted probabilities remained within the 0 to 1 range. Logistic regression was considered; however, the linear probability model was retained for ease of interpretation because the binary outcome was not rare and predicted probabilities remained within the 0 to 1 range. Because the error variance of a linear probability model is mechanically heteroskedastic, heteroskedasticity-robust (Huber–White) standard errors were used for the coefficient tests. Given the concurrent, single-time-point measurement of all variables, model coefficients are interpreted as cross-sectional associations rather than temporal predictions; the terms “associated with” and “differentiates” are therefore used in preference to “predicts” throughout the Results and Discussion. The analyses and the accompanying correlations are exploratory and hypothesis-generating; consequently, no formal family-wise correction for multiple comparisons was applied, and the reported *p*-values should be interpreted in this exploratory light, with primary emphasis placed on the magnitude of the effect sizes rather than on statistical significance alone. To evaluate whether the resilience typology was robust to the chosen cut-points rather than an artifact of them, sensitivity analyses were planned in which the barrier threshold (≥2 vs. ≥3 endorsed barriers) and the satisfaction threshold (E12 ≥ 4 vs. E12 = 5) were varied and the group comparisons re-examined; the outcome of these analyses is reported in Section 3.7.

### 2.7. Ethical Considerations

This study is an analysis of cross-sectional data originally collected by Elsayed and colleagues [6]. Ethical approval was obtained from the Ministry of Health Institutional Review Board, Saudi Arabia (IRB log No. 25–86E). All participants provided informed consent before data collection. The study was conducted in accordance with the principles of the Declaration of Helsinki. Data were de-identified prior to analysis.

## 3. Results

### 3.1. Resilience Group Classification (RQ1)

**Figure 1:**
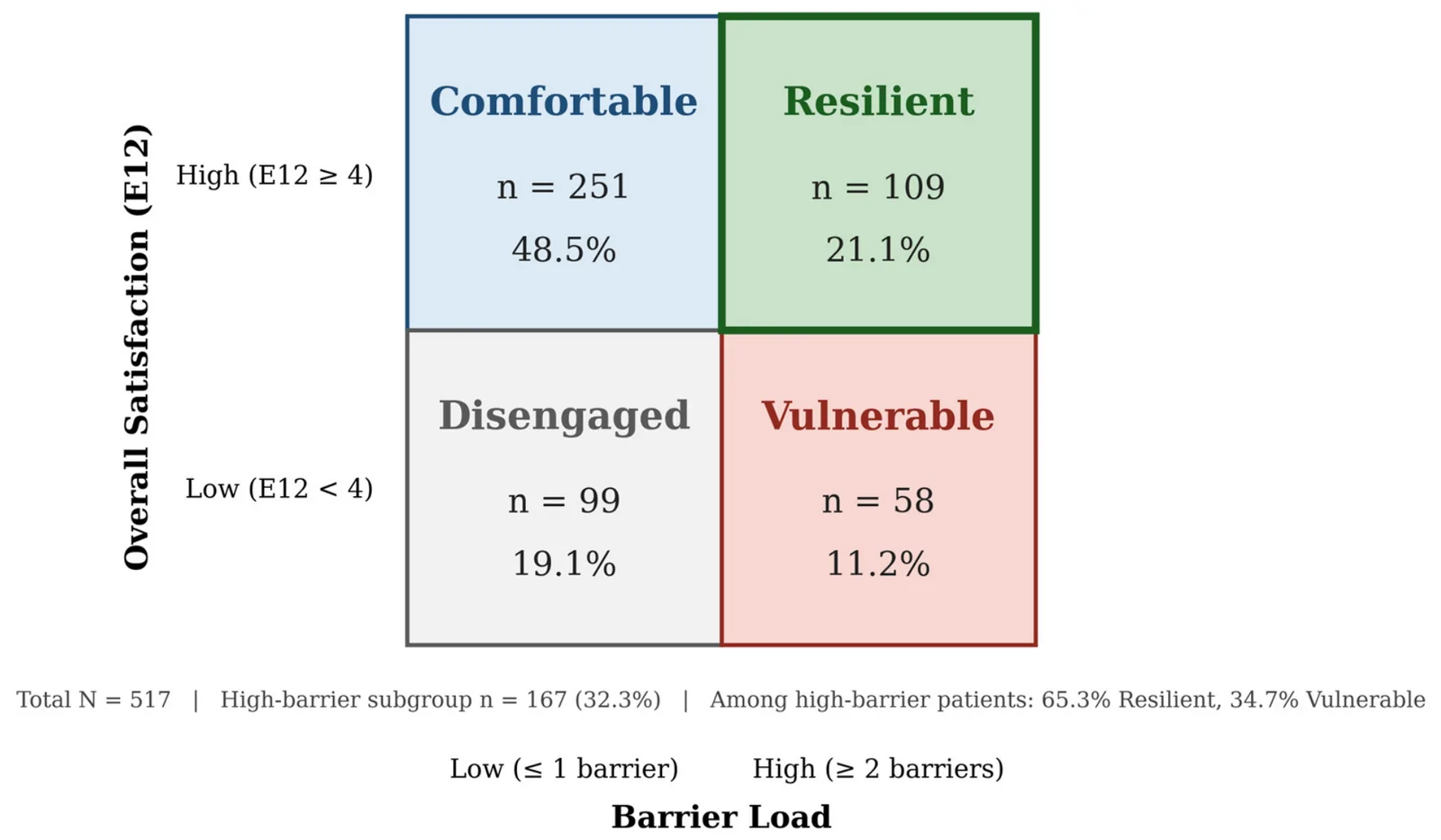
Satisfaction Resilience 2 × 2 Classification Matrix.

Of the 517 participants, 167 (32.3%) were classified as high-barrier (≥2 barriers endorsed) and 350 (67.7%) as low-barrier. When combined with the satisfaction indicator, this classification produced the four resilience groups shown in Table 1. The Comfortable group was the largest (*n* = 251, 48.5%), followed by Resilient (*n* = 109, 21.1%), Disengaged (*n* = 99, 19.1%), and Vulnerable (*n* = 58, 11.2%). Within the high-barrier subsample, 65.3% (109/167) were Resilient and 34.7% (58/167) were Vulnerable, indicating that the majority of patients who experienced barriers still maintained high satisfaction. Figure 1 displays the 2 × 2 classification matrix with absolute counts and percentages.

### 3.2. Group Differences Across All Variables

Table 2 presents the one-way ANOVA results across the four resilience groups for the main study variables. All ANOVAs were significant at *p* < 0.001. As expected from the group classification, the largest effects were observed for satisfaction (η^2^ = 0.690) and overall effectiveness (η^2^ = 0.172). It should be noted that the satisfaction item E12 is also a component of the 11-item Effectiveness composite and is the item used to define the satisfaction indicator; part of the between-group difference in the Effectiveness composite is therefore an arithmetic consequence of the classification rule rather than an independent empirical finding. To gauge the size of this dependency, the Effectiveness composite was recomputed after excluding E12 (a 10-item composite): the four-group difference remained large and statistically significant (η^2^ = 0.150, *p* < 0.001), and the Resilient–Vulnerable difference persisted (M = 4.06 vs. 3.63; d = 0.79), indicating that the composite difference is not driven solely by the definitional overlap with E12. This point is revisited in the interpretation of the functional-equivalence item E11 (Section 3.5) and in the Limitations. Beyond these variables, the largest effects were found for perceived functional equivalence item E11 (η^2^ = 0.133), motivation count (η^2^ = 0.089), provider trust E2 (η^2^ = 0.086), and the expression item E8 (η^2^ = 0.079). Comparison of the group means showed that Resilient patients scored higher than Vulnerable patients on all variables except barrier count, which was equivalent by definition of the group classification.

### 3.3. Resilient Versus Vulnerable: Identifying the Differentiators (RQ2)

The central comparison in this study was between the two high-barrier groups: Resilient and Vulnerable. These groups experienced similar levels of barriers but differed significantly in satisfaction. Their barrier counts were statistically equivalent (M = 2.24 vs. 2.26, t = 0.18, *p* = 0.857), confirming that the comparison focused on protective factors rather than differences in barrier exposure. Across all other variables, Resilient patients scored higher than Vulnerable patients. Motivation count was higher (M = 2.92 vs. 2.22; t = 4.07, *p* < 0.001, d = 0.695). Provider trust was higher (M = 4.05 vs. 3.57; t = 3.31, *p* = 0.001, d = 0.566). Similarly, overall effectiveness scores were higher among Resilient patients (M = 4.10 vs. 3.59; t = 5.06, *p* < 0.001, d = 0.869). The largest difference between the two groups was observed for perceived functional equivalence (E11: M = 3.73 vs. 2.36; t = 6.85, *p* < 0.001, d = 1.145).

**Figure 2:**
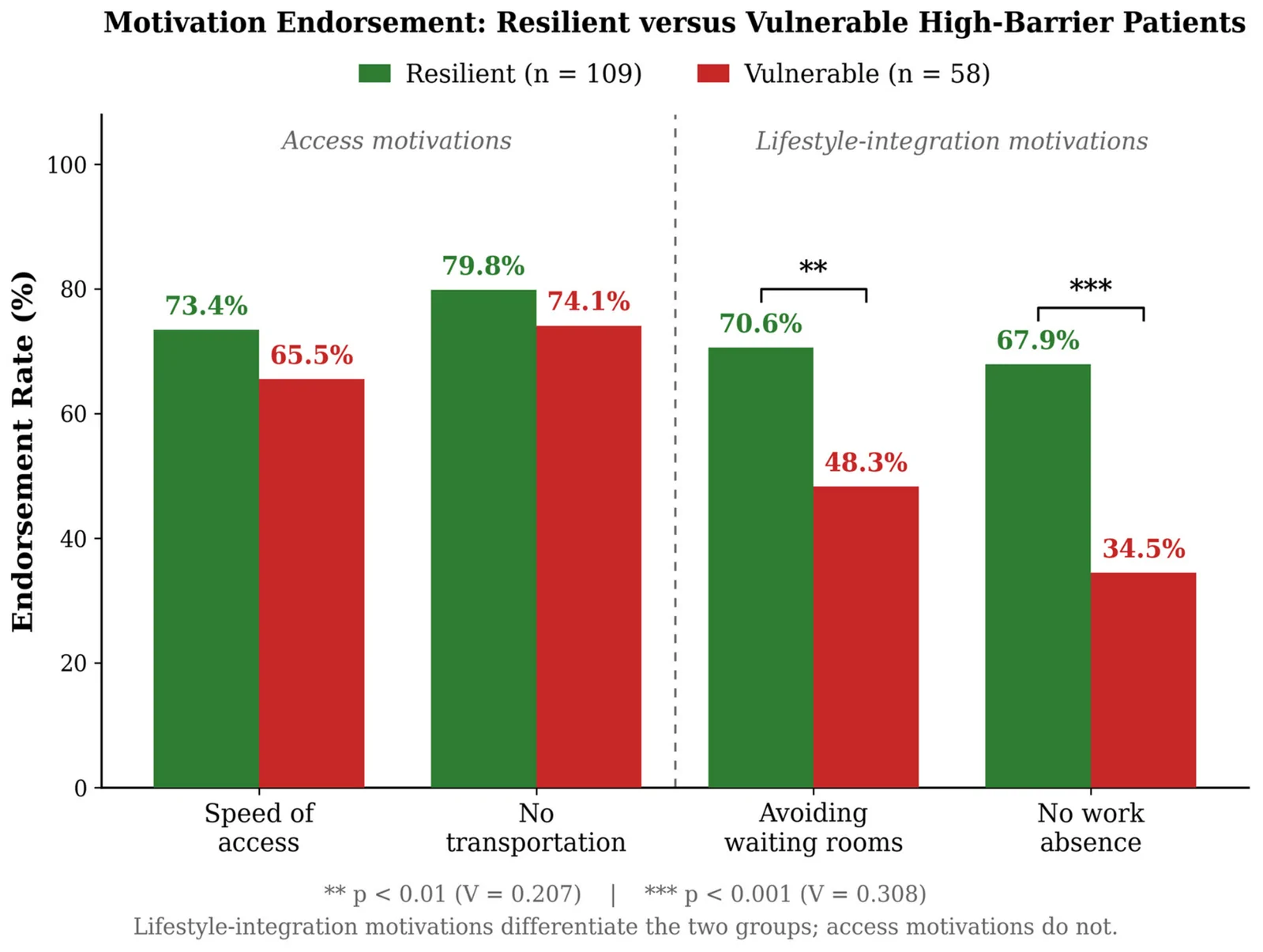
Motivation Endorsement Profiles: Resilient versus Vulnerable.

Table 3 examines the individual motivation and barrier items. The pattern of results was clear and supported the hypothesis that motivation type adds explanatory value beyond motivation count alone. The two lifestyle-integration motivations showed the strongest differences between the groups. Avoidance of work absence was endorsed by 67.9% of Resilient patients but only 34.5% of Vulnerable patients (χ^2^(1) = 15.84, *p* < 0.001, V = 0.308). Avoidance of waiting rooms was endorsed by 70.6% versus 48.3% (χ^2^(1) = 7.18, *p* = 0.007, V = 0.207). In contrast, the two access-related motivations did not clearly distinguish the groups. Speed of access was endorsed at 73.4% versus 65.5% (*p* = 0.376), and absence of transportation at 79.8% versus 74.1% (*p* = 0.519) (Figure 2). Among barriers, none of the four items significantly differentiated Resilient from Vulnerable patients, as expected, because barrier exposure was similar across both groups by definition.

### 3.4. Factors Associated with Resilience Within High-Barrier Patients

A linear probability model was estimated within the 167 high-barrier patients to identify factors independently associated with Resilient status (1 = Resilient, 0 = Vulnerable). The model included motivation count, provider trust (E2), and four demographic variables (gender, age, education, disease duration). The model explained 16.3% of the variance in resilience status (R^2^ = 0.163). Two variables were significantly associated with Resilient status (Table 4): motivation count (B = 0.121, SE = 0.029, t = 4.21, *p* < 0.001) and provider trust (B = 0.122, SE = 0.040, t = 3.04, *p* = 0.003). For each additional motivation endorsed, the probability of being Resilient (rather than Vulnerable) increased by approximately 12 percentage points. None of the demographic variables reached significance within this subsample.

Point-biserial correlations supported the differential motivation finding. The two lifestyle-integration motivations were significantly associated with resilience status: no work absence (r = 0.321, *p* < 0.001) and avoiding waiting rooms (r = 0.220, *p* = 0.004). In contrast, the two access motivations were not significantly associated with resilience status (speed of access: r = 0.082, *p* = 0.290; no transportation: r = 0.065, *p* = 0.403).

### 3.5. Perceived Functional Equivalence as the Strongest Single Differentiator

**Figure 3:**
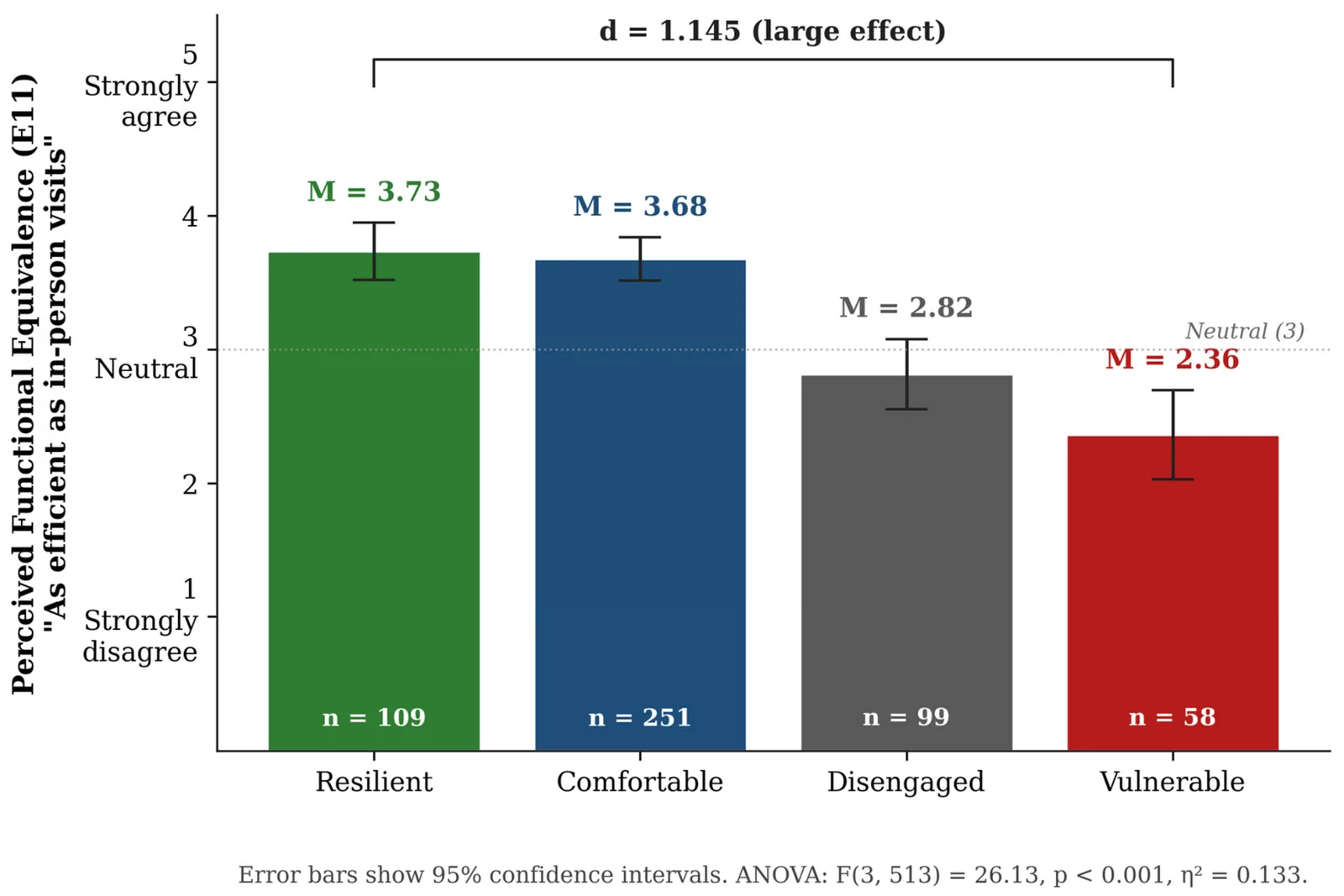
Perceived Functional Equivalence (E11) by Resilience Group.

The largest single effect-size difference between Resilient and Vulnerable patients was observed for the E11 item (“As efficient as in-person visits”), with d = 1.145. Resilient patients reported higher agreement with E11 at M = 3.73 (mild agreement), whereas Vulnerable patients averaged M = 2.36 (mild disagreement), representing a difference of 1.37 points on the 5-point scale. This effect was larger than the differences observed for motivation count (d = 0.695), provider trust (d = 0.566), and overall effectiveness (d = 0.869). The findings suggest that E11 may function as a particularly strong resilience anchor. Patients who believe VHHC is functionally equivalent to in-person care appear more likely to maintain satisfaction despite experiencing barriers, whereas those who do not hold this belief appear more vulnerable to dissatisfaction. Because E11 and the grouping item E12 belong to the same effectiveness battery, were collected by the same method from the same respondent at a single time point, the possibility of shared-method variance was examined. The correlation between E11 and E12 was moderate (r = 0.52), which is well below the near-collinearity observed between the two visual/audio equivalence items (E9–E10, r = 0.708) and indicates that E11 captures variance that is distinct from, rather than redundant with, the overall satisfaction item. Nonetheless, the large E11 effect may partly reflect a general positive-evaluation halo shared with E12 rather than a wholly independent threshold belief; this interpretive caveat is stated explicitly in the Limitations. Figure 3 shows the E11 distribution by resilience group.

### 3.6. Demographic Predictors of Resilience Group Membership (RQ3)

Across the full four-group classification, two demographic variables were associated with group membership. Age was associated with resilience group (χ^2^ (9) = 35.23, *p* < 0.001, V = 0.151), and education was similarly associated (χ^2^(6) = 23.77, *p* < 0.001, V = 0.152). Gender (*p* = 0.124) and disease duration (*p* = 0.942) were not associated with membership. The 25–35 age group showed the highest proportion of Resilient patients (31.9%), and postgraduate patients showed the highest proportion of Resilient patients (32.3%) compared with bachelor’s (17.8%) and high school (14.5%) patients.

These findings from the full sample should be interpreted carefully. The four-group classification combines two different dimensions: barrier exposure (low vs. high) and satisfaction outcome (low vs. high). As a result, demographic differences in group membership may reflect differences in barrier exposure, satisfaction outcomes, or both. To specifically examine resilience as defined in this study, maintaining satisfaction under barrier exposure, the analysis was restricted to 167 high-barrier patients, and demographic effects were re-examined. None of the four demographic variables were significantly associated with Resilient versus Vulnerable status within this subsample. These findings suggest that the demographic effects observed in the full sample mainly reflect differences in barrier exposure rather than differences in resilience itself. Once barrier exposure was held constant, resilience appeared to depend more on motivational and psychological resources than on demographic characteristics.

### 3.7. Sensitivity Analyses for the Classification Thresholds

Because the resilience typology depends on two dichotomization rules (barrier count ≥ 2 and E12 ≥ 4), a set of sensitivity analyses was conducted to test whether the Resilient–Vulnerable distinction is robust to the specific cut-points chosen rather than an artifact of them. Two alternative specifications were examined. First, the barrier threshold was raised from ≥2 to ≥3 endorsed barriers, restricting the high-barrier stratum to patients with the heaviest barrier load. Under this stricter definition, the ordering and direction of all group differences were preserved: Resilient patients continued to report higher motivation count, provider trust, and perceived functional equivalence than Vulnerable patients, and the two lifestyle-integration motivations remained the strongest item-level differentiators while the access motivations remained non-significant. Second, the satisfaction threshold was tightened from E12 ≥ 4 to E12 = 5 (strong agreement only). This specification likewise preserved the substantive pattern, with functional equivalence (E11) remaining the single largest differentiator between the high- and low-satisfaction high-barrier groups and motivation count and provider trust retaining their associations with high satisfaction. As expected, the stricter thresholds reduced cell sizes and therefore the precision of the estimates, but they did not reverse or eliminate any of the principal findings.

As a complementary check that does not rely on dichotomization, the overall satisfaction item (E12) was modeled as a continuous outcome using linear regression on barrier count, motivation count, and provider trust, together with a barrier × motivation interaction term, within the full sample. Barrier count was negatively associated with satisfaction, whereas motivation count and provider trust were positively associated with it, and the positive barrier × motivation interaction was consistent with the framework’s central premise: the negative association between barriers and satisfaction was attenuated among patients reporting more motivations. This continuous-outcome specification, which retains within-category variation discarded by the 2 × 2 typology, therefore reproduced the same substantive conclusion as the categorical analysis, supporting the interpretation that the resilience typology approximates a genuine moderation of the barrier–satisfaction relationship rather than an artifact of the chosen cut-points. The dichotomous typology is retained in the main analyses for its communicative and clinical accessibility, and the thresholds are treated throughout as conceptual classification rules rather than validated diagnostic anchors.

## 4. Discussion

This study introduces the concept of satisfaction resilience to VHHC literature and provides empirical support for three findings. First, the barrier–satisfaction relationship is heterogeneous: 65.3% of high-barrier patients maintained satisfaction, while 34.7% did not. Second, this heterogeneity was associated with motivational and psychological resources rather than with demographic characteristics. Third, the type of motivation played an important role: lifestyle-integration motivations differentiated Resilient from Vulnerable patients, whereas access motivations did not. Together these findings shift the practical orientation of telehealth satisfaction research from barrier elimination toward resilience building.

### 4.1. Lifestyle Integration as a Candidate Resilience Resource

The different effects of motivation type were particularly noticeable. Among Resilient and Vulnerable patients, who experienced similar levels of barriers, avoidance of work absence and avoidance of waiting rooms separated the groups by 33 and 22 percentage points respectively. In contrast, speed of access and avoidance of transportation, the two access-related motivations, did not separate the groups at all. This pattern was internally consistent. Access motivations reflect the practical advantages of VHHC, where individual consultations are faster or more convenient. In contrast, lifestyle-integration motivations reflect a structural fit between VHHC and the patient’s life pattern; the service supports working life, parenting obligations, and daily routines over time. These lifestyle-related motivations may therefore reflect a stronger and more persistent perceived fit between VHHC and patients’ everyday routines.

This finding has direct implications for VHHC communication design. Generic telehealth marketing often focuses on convenience and access, both of which were widely endorsed in our data but showed limited value in supporting resilience. VHHC program planners seeking to build long-term satisfaction should instead emphasize how VHHC fits into patients’ daily lives, including continuity of work, time efficiency for caregivers, and the reduction of overall time burden. The Saudi context is particularly well suited to this message. Given the scale of national digital health adoption and the large proportion of working-age adults in the present sample, the lifestyle-integration argument is particularly relevant. The finding is also consistent with Hepburn and colleagues’ updated COM-B systematic review [27], which identified motivation as the most consistent predictor of digital health adoption among older adults with chronic disease.

### 4.2. Functional Equivalence as a Candidate Resilience Threshold

The largest single effect observed in our data was for E11, the perceived functional equivalence item, with d = 1.145 between Resilient and Vulnerable patients. This effect was larger than those observed for motivation count, provider trust, and overall effectiveness. We interpret perceived functional equivalence as a threshold belief: patients who perceive VHHC as functionally equivalent to in-person care appear more likely to report high satisfaction despite barriers, whereas those who do not hold this belief appear more vulnerable to dissatisfaction. This effect appears to be structurally different from a graded predictor. The difference between an E11 score of 3.7 and 2.4 crosses the boundary between mild agreement and mild disagreement, which is the point at which the underlying belief changes direction.

This finding extends the Unified Theory of Acceptance and Use of Technology (UTAUT) [31] in a meaningful way. UTAUT identifies performance expectancy as an important determinant of technology acceptance. Our findings suggest that the same concept, expressed in patient terms as perceived functional equivalence, may also act as a *post-adoption resilience determinant*. Patients may initially adopt VHHC because they expect it to be effective. However, patients who continue to believe in its effectiveness appear more likely to maintain satisfaction despite barriers, while those who lose this belief become more vulnerable to dissatisfaction. This shift in viewing performance expectancy, from an adoption variable to a resilience variable, opens new opportunities for research on how functional-equivalence perceptions develop, strengthen, and change over time.

### 4.3. Demographics Are Associated with Sorting, Not Resilience

A subtle but important finding was that demographic variables were associated with four-group membership in the full sample but were not associated with Resilient versus Vulnerable status within the high-barrier subsample. The association of education and age with the four-group classification in the full sample appears to reflect differences in barrier exposure. Postgraduate patients may encounter more barriers because they evaluate VHHC more critically, and younger adults show a different pattern of motivation endorsement. However, once barrier exposure was held constant, demographic variables were no longer associated with resilience. At that point, resilience appeared to depend more on motivational and psychological factors than on demographic characteristics.

This finding has important practical implications for resilience-building interventions. Such interventions need not be targeted toward demographic groups. Instead, the same communication strategies—strengthening lifestyle-integration motivations, building functional equivalence beliefs, and reinforcing provider trust—may be effective across different age and education groups within the high-barrier population.

### 4.4. Trust as a Secondary Resilience Factor

Provider trust (E2) was the second significant predictor in the linear probability model (B = 0.122, *p* = 0.003), with an effect size comparable to motivation count. The Resilient group reported higher provider trust (M = 4.05) than the Vulnerable group (M = 3.57), representing a meaningful difference on a 5-point scale. The role of trust in resilience differs from its role in adoption. Trust does not eliminate barriers, as both groups experienced similar barriers. Instead, trust may influence how patients interpret those barriers. Patients with higher trust may view barriers as temporary or manageable aspects of the service, such as technology limitations that can improve over time, rather than as fundamental problems with virtual care. Previous telehealth literature has not clearly distinguished trust as an adoption driver from trust as a resilience resource [10]; our findings suggest that the distinction is meaningful and deserves further investigation, and they are consistent with evidence that adaptive patient–provider communication shapes chronic-illness self-management [32].

### 4.5. Implications for Multidisciplinary VHHC Delivery

VHHC in Saudi Arabia is delivered through multidisciplinary teams that include physicians, nurses, pharmacists, dietitians, and patient educators working through the same virtual platforms [1,2,28]. The satisfaction resilience framework has direct implications across these disciplines. Pharmacists conducting virtual medication reviews can present the encounter in lifestyle-integration terms (“this saves you a half-day off work”) rather than only in access terms. Nurses providing chronic disease education can focus on demonstrating functional equivalence by showing that the same teaching content, decision rules, and follow-up protocols apply regardless of whether care is delivered virtually or in person. Patient educators can focus their communication efforts on helping the high-barrier population build resilience rather than only trying to remove barriers.

The framework also supports better multidisciplinary care coordination. Different team members can be assigned different resilience-building roles: physicians can focus on demonstrating functional equivalence during consultations, pharmacists and nurses can reinforce lifestyle-integration messaging during follow-up contacts, and case managers can monitor patient satisfaction over time. The 21.1% Resilient and 11.2% Vulnerable proportions identified in this study provide a practical benchmark that VHHC programs can use to compare with their own patient populations and identify whether vulnerability is increasing over time.

### 4.6. Comparison with Previous Studies

The present findings both align with and extend the existing telehealth satisfaction literature. The high overall satisfaction observed here is consistent with large-sample cross-sectional studies of chronic disease patients: Hendy and colleagues reported that roughly two-thirds of 1070 chronic disease patients were satisfied with telehealth [16], a proportion close to the 65.3% Resilient share observed among high-barrier patients in this study, and Spaulding and colleagues similarly documented broadly favorable telehealth satisfaction in a large United States cross-sectional survey [33], with comparably high satisfaction reported for telemedicine in other service contexts [34,35]. At the system level, the Seha Virtual Hospital outcome analysis reported that around 86% of outpatients recorded satisfactory outcomes in 2022 and 2024 [1], and a nationwide Saudi cross-sectional study found that more than 90% of respondents preferred virtual over in-person appointments [36], corroborating the high baseline satisfaction against which the present resilience framework is set.

Where earlier work diverges from the present study is in its treatment of demographic characteristics. Several cross-sectional analyses have identified age, sex, and education as statistically significant correlates of telehealth satisfaction—Hendy et al. reported lower satisfaction among older patients and higher satisfaction among more educated patients [16], and Lee and Nam found that telehealth utilization and experience varied across sociodemographic groups in a national survey [37]. The present study reproduces this pattern in the full sample, where age and education were associated with four-group membership, but shows that once barrier exposure is held constant these demographic associations disappear, and motivational and psychological resources become the salient differentiators. This is an important qualification of the demographic-determinant narrative: demographic variables appear to sort patients into differing levels of barrier exposure rather than to govern resilience under barriers directly.

The prominence of perceived functional equivalence as the single strongest differentiator is consistent with the broader evidence that telehealth achieves clinical and experiential outcomes comparable to in-person care. Systematic reviews and meta-analyses have repeatedly reported small or non-clinically meaningful differences between telehealth and in-person care across chronic conditions [12,27], and primary-care syntheses have found broadly comparable patient satisfaction between synchronous telemedicine and face-to-face visits [38]. Nationwide Saudi data likewise show favorable patient comparisons of convenience, quality of interaction, and satisfaction between virtual and in-person consultations [39]. The present study adds a patient-perception dimension to this equivalence literature by showing that it is the patient’s own belief in functional equivalence, rather than objective equivalence alone, that most strongly distinguishes patients who remain satisfied under barriers from those who do not. Likewise, the salience of motivation echoes recent COM-B applications to digital health adoption, in which motivational factors outweighed opportunity constraints among older adults with chronic disease [27], while the differentiation between lifestyle-integration and access motivations is, to our knowledge, novel and not previously reported in the telehealth satisfaction literature. Finally, the provider-trust finding is consistent with work identifying trust, communication, and workflow integration as determinants of telehealth experience and satisfaction [8,18,40], while reframing trust specifically as a resource that shapes how existing barriers are interpreted rather than only as an adoption driver. Taken together, the study is continuous with prior evidence on high satisfaction and functional equivalence, but departs from it by demonstrating that the barrier–satisfaction relationship is heterogeneous and by identifying the motivational and belief-based resources associated with preserved satisfaction under barrier exposure.

## 5. Limitations of the Study

Several limitations should be acknowledged. The cross-sectional design prevents causal inference about the direction of effects between motivation, trust, functional equivalence, and satisfaction. The application of the resilience construct to cross-sectional data is a conceptual adaptation rather than a direct operationalization of the traditional psychological definition, which requires longitudinal measurement of recovery over time. In the psychological and chronic-disease literature, resilience is understood as a dynamic process of adaptation and recovery that unfolds over time in response to adversity, and it is therefore properly measured through repeated observation. The present study deliberately uses “satisfaction resilience” in a narrower, explicitly cross-sectional sense—the maintenance of high satisfaction in the concurrent presence of multiple barriers at a single time point—and this state-based usage is intended as a descriptive label for a pattern of preserved satisfaction under barrier exposure, not as a claim that a longitudinal resilience process has been observed. Readers should interpret every use of the term in this manuscript accordingly, and the framework’s dynamic properties remain to be established in prospective work. The present findings capture resilience as a concurrent state—high satisfaction alongside multiple barriers at a single time point—and cannot determine whether Resilient patients would sustain this state following further barrier accumulation, nor whether Vulnerable patients would recover over time. Longitudinal designs are required to test the dynamic properties of satisfaction resilience as defined here. The 2 × 2 classification uses binary thresholds (≥2 barriers, ≥4 satisfaction) that simplify continuous variables and may obscure boundary cases; sensitivity analyses using alternative cutoffs would strengthen the framework. The motivation and barrier items were multi-select categories rather than validated psychometric scales, which limits item-level psychometric evaluation. Social desirability bias may inflate motivation endorsement and suppress barrier reporting in self-report surveys. The Vulnerable subgroup (*n* = 58) was the smallest of the four groups, which reduces statistical power for further subdivision. Geographic concentration in the Central Region (88% of the sample) limits generalizability to other Saudi regions and similar Gulf populations. Beyond geographic concentration, the recruitment strategy itself is a source of selection bias: participants were recruited online through digital health platforms and healthcare facility networks for a study evaluating a digital health service, which plausibly oversamples patients who are already comfortable with, and favorably disposed toward, virtual care. This recruitment-channel selection mechanism may bias the reported group proportions—inflating satisfaction and the 65.3% Resilient share among high-barrier patients while deflating estimated barrier exposure—relative to the full population of VHHC users, and more so relative to the broader chronic disease population in Saudi Arabia. The reported prevalences should therefore be read as descriptive of this convenience sample rather than as population estimates. A further interpretive limitation concerns the effectiveness composite and the functional-equivalence item: because the satisfaction item E12 is embedded in the effectiveness battery and is also the grouping variable, part of the Effectiveness between-group difference is definitionally tied to the classification, and the large E11 effect may partly reflect shared-method variance and a positive-evaluation halo shared with E12 rather than a wholly distinct threshold belief, notwithstanding the moderate E11–E12 correlation reported. Several potentially important variables were neither measured nor adjusted for and may act as common causes of both barrier endorsement and satisfaction, including chronic disease type, disease severity or comorbidity burden, digital and health literacy, socioeconomic status, and prior experience with telehealth [41]; the associations reported in Table 4, which adjust only for four demographic covariates, are therefore subject to residual confounding. Chronic disease status was captured by self-report at the time of survey completion, without independent clinical confirmation, and the multi-select motivation and barrier items were recoded from semicolon-delimited strings into binary indicators using a deterministic string-matching procedure against the fixed, pre-specified answer options; because respondents selected from closed options rather than entering free text, matching was exact and did not require adjudication of free-text variants, but this closed-option format also precludes item-level psychometric evaluation. The regression model accounted for a modest share of the variance in resilience status (R^2^ = 0.163), indicating that additional, unmeasured determinants of satisfaction resilience remain to be identified; the predictive performance of the model should not be overinterpreted, and the coefficients are best read as cross-sectional associations rather than as evidence of predictive accuracy. Finally, as an analysis of an existing dataset, the study was constrained to the variables originally collected and could not incorporate measures that were not part of the parent instrument. The newly proposed satisfaction resilience framework has not yet been externally validated. Future work should pursue prospective longitudinal designs that track resilience stability over time, test whether resilience-building interventions can shift Vulnerable patients toward Resilient status, incorporate the confounders listed above, and replicate the framework in other regions of Saudi Arabia and in different healthcare systems to establish its generalizability beyond the present setting.

## 6. Conclusions

The results of this study show that the barrier–satisfaction relationship in VHHC is not uniform, and they answer the three research questions posed at the outset. Addressing RQ1, the four satisfaction resilience groups were unevenly distributed—Comfortable (48.5%), Resilient (21.1%), Disengaged (19.1%), and Vulnerable (11.2%)—and differed markedly on satisfaction, perceived effectiveness, functional equivalence, motivation count, and provider trust, but were essentially equivalent on barrier count among the high-barrier groups. Addressing RQ2, among 167 chronic disease patients facing equivalent high-barrier loads, 65.3% maintained satisfaction (Resilient) while 34.7% did not (Vulnerable), and the factors distinguishing them were motivational and psychological rather than demographic: lifestyle-integration motivations (avoiding work absence, avoiding waiting rooms) differentiated the groups by 22 to 33 percentage points, whereas access motivations (speed, transportation) did not differentiate at all; perceived functional equivalence with in-person care produced the largest single effect (d = 1.145), consistent with its possible role as a threshold belief associated with preserved satisfaction under barrier conditions, and provider trust was independently associated with resilience. Addressing RQ3, demographic variables (age and education) were associated with group membership across the full sample but were not associated with Resilient versus Vulnerable status once barrier exposure was held constant, indicating that demographics are associated with sorting into barrier exposure rather than with resilience itself. Within the high-barrier population, no demographic variable was significantly associated with resilience.

These findings shift the practical orientation of VHHC satisfaction research. VHHC programs seeking to improve satisfaction outcomes do not need to focus only on removing barriers, especially because some barriers are inherent to remote care. They can also build resilience among the substantial barrier-exposed population by emphasizing lifestyle integration rather than general convenience, by building functional equivalence beliefs through evidence-based communication, and by drawing on existing provider trust to shape how patients interpret those barriers. As the Seha Virtual Hospital and the broader Saudi VHHC infrastructure continue to expand under Vision 2030, satisfaction resilience offers a multidisciplinary framework that can be applied across physician, nursing, pharmacy, and education services. The framework may also help teams identify patients who need additional resilience-building support and distinguish them from those whose satisfaction is already stable.

## Figures and Tables

**Table 1:** Satisfaction Resilience Group Classification and Group Profiles (N = 517).

Group	*n*	%	Barrier Count	Motivation Count	Satisfaction (E12)	Trust (E2)	Effectiveness	Female %	>5 Years %
Resilient	109	21.1	2.24	2.92	4.16	4.05	4.10	68.8	55.0
Vulnerable	58	11.2	2.26	2.22	2.74	3.57	3.59	56.9	56.9
Comfortable	251	48.5	1.00	2.38	4.26	4.20	4.07	72.9	58.6
Disengaged	99	19.1	1.00	1.85	2.80	3.58	3.38	69.7	57.6

**Table 2:** One-Way ANOVA Across the Four Resilience Groups (N = 517).

Variable	Resilient	Vulnerable	Comfortable	Disengaged	F	*p*	η^2^
Satisfaction (E12)	4.16	2.74	4.26	2.80	381.04	<0.001	0.690
Effectiveness	4.10	3.59	4.07	3.38	35.51	<0.001	0.172
E11 Functional Equivalence	3.73	2.36	3.68	2.82	26.13	<0.001	0.133
Motivation count	2.92	2.22	2.38	1.85	16.67	<0.001	0.089
Trust (E2)	4.05	3.57	4.20	3.58	16.01	<0.001	0.086
E8 Expression	4.19	3.57	4.02	3.33	14.71	<0.001	0.079

Notes: Cell entries are means. All F-tests significant at *p* < 0.001. Effect sizes follow Cohen’s conventions: η^2^ ≥ 0.01 small, ≥0.06 medium, ≥0.14 large.

**Table 3:** Item-Level Comparison of Resilient (*n* = 109) and Vulnerable (*n* = 58) Groups.

Domain	Item	Resilient %	Vulnerable %	χ^2^	*p*	Cramér’s V	Differentiates?
Motivation	Speed of access	73.4	65.5	0.78	0.376	0.069	No
Motivation	No transportation needed	79.8	74.1	0.42	0.519	0.050	No
Motivation	Avoiding waiting rooms	70.6	48.3	7.18	0.007	0.207	Yes
Motivation	No work absence	67.9	34.5	15.84	<0.001	0.308	Yes
Barrier	Accuracy doubt	79.8	91.4	2.93	0.087	0.132	No
Barrier	Quality concern	84.4	87.9	0.15	0.699	0.030	No
Barrier	Privacy worry	35.8	32.8	0.05	0.826	0.017	No

Notes: Cell entries are endorsement percentages. “Differentiates?” indicates whether the item significantly distinguishes Resilient from Vulnerable at *p* < 0.05. Lifestyle-integration motivations differentiate; access motivations and barrier items do not.

**Table 4:** Linear Probability Model Predicting Resilient Status Within High-Barrier Patients (*n* = 167).

Predictor	B	SE	t	*p*	Interpretation
Motivation count	0.121	0.029	4.21	<0.001	Significant
Provider trust (E2)	0.122	0.040	3.04	0.003	Significant
Gender	−0.041	0.075	−0.55	0.583	ns
Age	0.029	0.040	0.73	0.469	ns
Education	0.046	0.061	0.75	0.452	ns
Disease duration	0.012	0.073	0.16	0.872	ns

Notes: Outcome variable: 1 = Resilient, 0 = Vulnerable. Model R^2^ = 0.163, F(6, 160) = 5.19, *p* < 0.001. ns = not significant. Demographic variables were coded as indicators (gender) or ordinal (age, education, duration).

## Data Availability

The data presented in this study are available on reasonable request from the corresponding author. The data are not publicly available due to ethical and privacy restrictions related to human-participant data, the terms of informed consent, and institutional approval requirements. Any data sharing will be subject to approval by the relevant institutional ethics authority and will be limited to de-identified data necessary to verify the study findings.
